# {1-[(2-Oxidonaphthalen-1-yl)methylidene]thiosemicarbazidato-κ^3^ *N* ^1^,*O*,*S*}diphenyl­tin(IV)

**DOI:** 10.1107/S1600536810054024

**Published:** 2011-01-08

**Authors:** Jichun Cui, Hong Ruan, Yanling Qiao, Handong Yin

**Affiliations:** a College of Chemistry and Chemical Engineering, Liaocheng University, Shandong 252059, People’s Republic of China

## Abstract

The asymmetric unit of the title compound, [Sn(C_6_H_5_)_2_(C_12_H_9_N_3_OS)], contains two independent mol­ecules with almost identical configurations. In each mol­ecule, the Sn^IV^ atom is coordinated by O, N and S atoms from a (2-oxido-1-naph­thaldehyde)­thio­semicarbazonato ligand and two C atoms from phenyl rings in a distorted trigonal–bipyramidal geometry. Weak inter­molecular N—H⋯O and N—H⋯S hydrogen bonds link four mol­ecules into a centrosymmetric tetra­mer. The crystal packing exhibits short inter­molecular S⋯S contacts of 3.335 (3) Å.

## Related literature

For related organotin(IV) complexes with salicyl­aldehyde­thio­semicarbazones, see: Sarma *et al.* (2007 ▶).
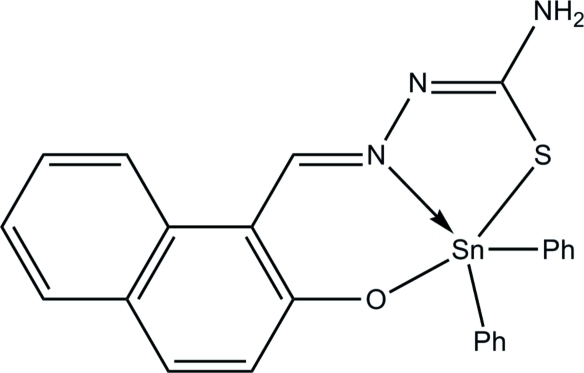


## Experimental

### 

#### Crystal data

[Sn(C_6_H_5_)_2_(C_12_H_9_N_3_OS)]
*M* *_r_* = 516.17
Triclinic, 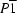

*a* = 10.0228 (14) Å
*b* = 10.9676 (16) Å
*c* = 19.732 (3) Å
α = 89.880 (2)°
β = 88.696 (2)°
γ = 85.435 (1)°
*V* = 2161.6 (5) Å^3^
*Z* = 4
Mo *K*α radiation
μ = 1.30 mm^−1^
*T* = 298 K
0.23 × 0.18 × 0.12 mm

#### Data collection

Bruker SMART 1000 diffractometer
Absorption correction: multi-scan (*SADABS*; Bruker, 2001 ▶) *T*_min_ = 0.754, *T*_max_ = 0.860
10805 measured reflections
7339 independent reflections
5240 reflections with *I* > 2σ(*I*)
*R*_int_ = 0.032

#### Refinement

*R*[*F* ^2^ > 2σ(*F* ^2^)] = 0.046
*wR*(*F* ^2^) = 0.133
*S* = 1.00
7339 reflections
541 parameters
H-atom parameters constrained
Δρ_max_ = 1.56 e Å^−3^
Δρ_min_ = −0.79 e Å^−3^

### 

Data collection: *SMART* (Bruker, 2007 ▶); cell refinement: *SAINT* (Bruker, 2007 ▶); data reduction: *SAINT*; program(s) used to solve structure: *SHELXS97* (Sheldrick, 2008 ▶); program(s) used to refine structure: *SHELXL97* (Sheldrick, 2008 ▶); molecular graphics: *SHELXTL* (Sheldrick, 2008 ▶); software used to prepare material for publication: *SHELXTL*.

## Supplementary Material

Crystal structure: contains datablocks I, global. DOI: 10.1107/S1600536810054024/cv5008sup1.cif

Structure factors: contains datablocks I. DOI: 10.1107/S1600536810054024/cv5008Isup2.hkl

Additional supplementary materials:  crystallographic information; 3D view; checkCIF report

## Figures and Tables

**Table 1:** Hydrogen-bond geometry (Å, °)

*D*—H⋯*A*	*D*—H	H⋯*A*	*D*⋯*A*	*D*—H⋯*A*
N6—H6*B*⋯O1^i^	0.86	2.35	3.209 (6)	177
N3—H3*A*⋯S2^ii^	0.86	2.87	3.522 (6)	134

Symmetry codes: (i) 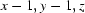; (ii) 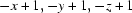.

**Fig. 1.**
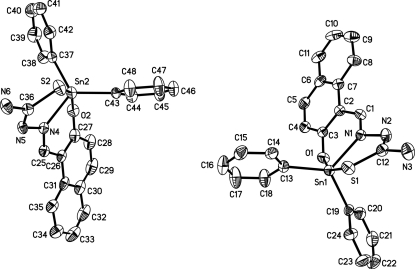
The asymmetric unit of the title compound, showing 50% probability displacement ellipsoids. H atoms have been omitted for clarity.

**Fig. 2.**
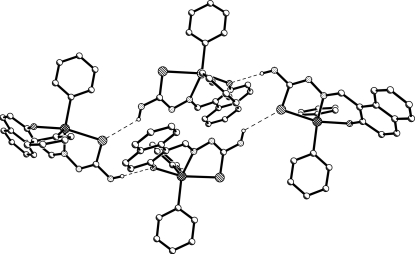
The hydrogen-bonded (dashed lines) tetramer in the title compound. H atoms not involved in hydrogen bonding have been omitted for clarity.

## supplementary crystallographic information

### Comment

Recently, some organotin(IV) complexes of salicylaldehyde- thiosemicarbazones have been reported (Sarma *et al.*, 2007). As an extension of the work on the organotin(IV) thiosemicarbazones chemistry, the title compound, (I), is reported here (Fig. 1).

In both independent molecules of the title compound, (I), the Sn atom has distorted trigonal-bipyramidal geometry, with atoms O and S in axial positions [O1—Sn1—S1 = 157.99 (11)°, O2—Sn2—S2 = 157.71 (13)°] and the atoms C, C and N in equatorial positions. The sum of the equatorial angles is 359.96 ° and 359.78 °, indicating approximate coplanarity for these atoms in two molecules. The Sn1—N1 (Sn2—N4) bond length is 2.175 (4) Å (2.185 (5) Å) close to the sum of the non-polar covalent radii 2.15 Å, indicating a strong Sn—N interaction.

Weak intermolecular N—H···O and N—H···S hydrogen bonds (Table 1) link four molecules into centrosymmetric tetramer (Fig. 2). The crystal packing exhibits short intermolecular S···S contacts of 3.335 (3) Å.

### Experimental

The reaction was carried out under nitrogen atmosphere. 2-hydroxy-1-naphthaldehydethiosemicarbazone (1 mmol) and sodium ethoxide (1.1 mmol) were added to the solution of benzene(30 ml) in a Schlenk flask and stirred for 0.5 h. Diphenyltin dichloride (1 mmol) was then added to the reactor and the reaction mixture was stirredfor 4 h at 313 K. The resulting clear solution was evaporated under vacuum. The product was crystallized from a mixture of dichloromethane/methanol (1:1) to yield orange blocks of the title compound (yield 73%).

### Refinement

All H atoms were positioned geometrically (C—H 0.93 Å, N—H 0.86 Å), and refined as riding on their parent atoms, with *U*_iso_(H) = 1.2 *U*_eq_(C, N).

### Crystal data

**Table:**

[Sn(C_6_H_5_)_2_(C_12_H_9_N_3_OS)(C_6_H_5_)_2_]	*Z* = 4
*M**_r_* = 516.17	*F*(000) = 1032
Triclinic, *P*1	*D*_x_ = 1.586 Mg m^−^^3^
*a* = 10.0228 (14) Å	Mo *K*α radiation, λ = 0.71073 Å
*b* = 10.9676 (16) Å	Cell parameters from 4687 reflections
*c* = 19.732 (3) Å	θ = 2.3–26.9°
α = 89.880 (2)°	µ = 1.30 mm^−^^1^
β = 88.696 (2)°	*T* = 298 K
γ = 85.435 (1)°	Block, orange
*V* = 2161.6 (5) Å^3^	0.23 × 0.18 × 0.12 mm

### Data collection

**Table:**

Bruker SMART 1000 diffractometer	7339 independent reflections
Radiation source: fine-focus sealed tube	5240 reflections with *I* > 2σ(*I*)
graphite	*R*_int_ = 0.032
phi and ω scans	θ_max_ = 25.0°, θ_min_ = 1.9°
Absorption correction: multi-scan (*SADABS*; Bruker, 2001)	*h* = −11→11
*T*_min_ = 0.754, *T*_max_ = 0.860	*k* = −13→12
10805 measured reflections	*l* = −23→17

### Refinement

**Table:**

Refinement on *F*^2^	Primary atom site location: structure-invariant direct methods
Least-squares matrix: full	Secondary atom site location: difference Fourier map
*R*[*F*^2^ > 2σ(*F*^2^)] = 0.046	Hydrogen site location: inferred from neighbouring sites
*wR*(*F*^2^) = 0.133	H-atom parameters constrained
*S* = 1.00	*w* = 1/[σ^2^(*F*_o_^2^) + (0.0739*P*)^2^] where *P* = (*F*_o_^2^ + 2*F*_c_^2^)/3
7339 reflections	(Δ/σ)_max_ = 0.001
541 parameters	Δρ_max_ = 1.56 e Å^−^^3^
0 restraints	Δρ_min_ = −0.79 e Å^−^^3^

### Fractional atomic coordinates and isotropic or equivalent isotropic displacement parameters (Å^2^)

**Table:**

	*x*	*y*	*z*	*U*_iso_*/*U*_eq_	
Sn1	0.94444 (4)	0.74053 (3)	0.412798 (19)	0.03969 (14)	
Sn2	0.00729 (4)	0.23449 (3)	0.106121 (19)	0.04414 (14)	
N1	0.8748 (4)	0.8873 (4)	0.4820 (2)	0.0389 (10)	
N2	0.9313 (5)	0.8903 (4)	0.5463 (2)	0.0492 (12)	
N3	1.0343 (6)	0.7806 (5)	0.6313 (3)	0.0689 (16)	
H3A	1.0376	0.8470	0.6540	0.083*	
H3B	1.0664	0.7121	0.6479	0.083*	
N4	0.0984 (4)	0.0635 (4)	0.0630 (2)	0.0414 (11)	
N5	0.0689 (5)	−0.0488 (4)	0.0921 (2)	0.0462 (12)	
N6	−0.0081 (6)	−0.1457 (4)	0.1860 (3)	0.0641 (15)	
H6A	−0.0010	−0.2137	0.1641	0.077*	
H6B	−0.0367	−0.1437	0.2274	0.077*	
O1	0.8805 (4)	0.8727 (4)	0.33971 (18)	0.0475 (10)	
O2	0.0506 (4)	0.2965 (4)	0.0086 (2)	0.0547 (11)	
S1	0.97185 (17)	0.64262 (14)	0.52905 (8)	0.0507 (4)	
S2	0.0102 (2)	0.08899 (15)	0.20601 (8)	0.0600 (5)	
C1	0.7892 (6)	0.9790 (5)	0.4688 (3)	0.0454 (14)	
H1	0.7695	1.0353	0.5035	0.055*	
C2	0.7216 (5)	1.0023 (5)	0.4060 (3)	0.0403 (13)	
C3	0.7693 (5)	0.9484 (5)	0.3446 (3)	0.0412 (13)	
C4	0.7010 (6)	0.9777 (6)	0.2835 (3)	0.0507 (15)	
H4	0.7317	0.9409	0.2431	0.061*	
C5	0.5912 (6)	1.0589 (6)	0.2838 (3)	0.0584 (17)	
H5	0.5494	1.0775	0.2430	0.070*	
C6	0.5381 (6)	1.1164 (6)	0.3440 (3)	0.0533 (16)	
C7	0.6038 (6)	1.0868 (5)	0.4063 (3)	0.0475 (14)	
C8	0.5456 (6)	1.1439 (6)	0.4660 (4)	0.0601 (17)	
H8	0.5850	1.1271	0.5076	0.072*	
C9	0.4336 (7)	1.2223 (6)	0.4635 (4)	0.072 (2)	
H9	0.3978	1.2573	0.5035	0.086*	
C10	0.3716 (7)	1.2512 (7)	0.4030 (5)	0.080 (2)	
H10	0.2961	1.3062	0.4022	0.096*	
C11	0.4231 (6)	1.1975 (7)	0.3439 (4)	0.070 (2)	
H11	0.3806	1.2154	0.3033	0.084*	
C12	0.9781 (6)	0.7837 (6)	0.5686 (3)	0.0472 (14)	
C13	0.8249 (6)	0.6076 (5)	0.3716 (3)	0.0440 (14)	
C14	0.7493 (6)	0.5338 (6)	0.4118 (4)	0.0553 (16)	
H14	0.7450	0.5456	0.4585	0.066*	
C15	0.6798 (7)	0.4425 (6)	0.3832 (4)	0.0681 (19)	
H15	0.6307	0.3927	0.4107	0.082*	
C16	0.6844 (9)	0.4264 (7)	0.3135 (5)	0.085 (3)	
H16	0.6364	0.3669	0.2940	0.102*	
C17	0.7581 (10)	0.4965 (8)	0.2743 (4)	0.099 (3)	
H17	0.7624	0.4834	0.2277	0.119*	
C18	0.8277 (8)	0.5881 (7)	0.3016 (4)	0.075 (2)	
H18	0.8766	0.6368	0.2733	0.090*	
C19	1.1504 (5)	0.7491 (5)	0.3860 (3)	0.0424 (13)	
C20	1.1968 (6)	0.8478 (6)	0.3512 (3)	0.0550 (16)	
H20	1.1373	0.9127	0.3384	0.066*	
C21	1.3335 (8)	0.8495 (8)	0.3353 (4)	0.076 (2)	
H21	1.3650	0.9161	0.3124	0.091*	
C22	1.4206 (8)	0.7535 (10)	0.3533 (4)	0.087 (3)	
H22	1.5112	0.7554	0.3424	0.104*	
C23	1.3769 (7)	0.6546 (9)	0.3871 (4)	0.083 (2)	
H23	1.4369	0.5891	0.3985	0.099*	
C24	1.2427 (6)	0.6534 (6)	0.4039 (3)	0.0595 (17)	
H24	1.2131	0.5872	0.4278	0.071*	
C25	0.1833 (6)	0.0530 (6)	0.0113 (3)	0.0483 (15)	
H25	0.2173	−0.0257	−0.0004	0.058*	
C26	0.2289 (6)	0.1518 (6)	−0.0291 (3)	0.0461 (14)	
C27	0.1601 (7)	0.2678 (6)	−0.0279 (3)	0.0548 (16)	
C28	0.2088 (8)	0.3617 (7)	−0.0708 (4)	0.072 (2)	
H28	0.1634	0.4390	−0.0713	0.087*	
C29	0.3204 (9)	0.3383 (8)	−0.1105 (4)	0.082 (3)	
H29	0.3500	0.4008	−0.1375	0.099*	
C30	0.3926 (7)	0.2236 (8)	−0.1124 (3)	0.0650 (19)	
C31	0.3494 (6)	0.1270 (7)	−0.0721 (3)	0.0550 (17)	
C32	0.5141 (9)	0.2039 (11)	−0.1522 (4)	0.088 (3)	
H32	0.5429	0.2671	−0.1789	0.105*	
C33	0.5871 (9)	0.0951 (11)	−0.1514 (4)	0.093 (3)	
H33	0.6674	0.0848	−0.1761	0.111*	
C34	0.5426 (7)	−0.0016 (8)	−0.1138 (3)	0.076 (2)	
H34	0.5912	−0.0774	−0.1148	0.091*	
C35	0.4265 (7)	0.0149 (7)	−0.0748 (3)	0.0631 (18)	
H35	0.3986	−0.0505	−0.0496	0.076*	
C36	0.0259 (6)	−0.0414 (5)	0.1551 (3)	0.0438 (13)	
C37	−0.2025 (6)	0.2693 (5)	0.0914 (3)	0.0454 (14)	
C38	−0.2527 (7)	0.3028 (6)	0.0285 (3)	0.0628 (18)	
H38	−0.1946	0.3020	−0.0089	0.075*	
C39	−0.3868 (8)	0.3373 (7)	0.0203 (4)	0.085 (2)	
H39	−0.4177	0.3603	−0.0223	0.101*	
C40	−0.4757 (8)	0.3378 (7)	0.0752 (5)	0.083 (2)	
H40	−0.5662	0.3607	0.0696	0.100*	
C41	−0.4287 (7)	0.3043 (7)	0.1376 (4)	0.074 (2)	
H41	−0.4872	0.3042	0.1748	0.089*	
C42	−0.2932 (7)	0.2704 (6)	0.1452 (4)	0.0622 (18)	
H42	−0.2626	0.2477	0.1879	0.075*	
C43	0.1128 (5)	0.3726 (5)	0.1536 (3)	0.0421 (13)	
C44	0.1418 (7)	0.4771 (6)	0.1197 (3)	0.0599 (17)	
H44	0.1222	0.4854	0.0740	0.072*	
C45	0.2000 (7)	0.5706 (6)	0.1527 (3)	0.0665 (19)	
H45	0.2189	0.6405	0.1287	0.080*	
C46	0.2299 (8)	0.5615 (6)	0.2196 (4)	0.068 (2)	
H46	0.2675	0.6250	0.2415	0.082*	
C47	0.2035 (9)	0.4571 (7)	0.2540 (4)	0.084 (3)	
H47	0.2248	0.4493	0.2995	0.100*	
C48	0.1457 (8)	0.3634 (6)	0.2221 (4)	0.069 (2)	
H48	0.1285	0.2934	0.2464	0.083*	

### Atomic displacement parameters (Å^2^)

**Table:**

	*U*^11^	*U*^22^	*U*^33^	*U*^12^	*U*^13^	*U*^23^
Sn1	0.0424 (2)	0.0429 (2)	0.0336 (2)	−0.00235 (17)	−0.00036 (16)	0.00203 (17)
Sn2	0.0562 (3)	0.0419 (2)	0.0352 (2)	−0.00743 (18)	−0.00820 (18)	0.00630 (17)
N1	0.043 (3)	0.043 (3)	0.031 (3)	−0.005 (2)	−0.0026 (19)	0.003 (2)
N2	0.064 (3)	0.052 (3)	0.032 (3)	−0.009 (2)	−0.010 (2)	0.004 (2)
N3	0.095 (4)	0.071 (4)	0.042 (3)	−0.006 (3)	−0.020 (3)	0.005 (3)
N4	0.049 (3)	0.047 (3)	0.030 (3)	−0.013 (2)	−0.001 (2)	0.004 (2)
N5	0.065 (3)	0.036 (3)	0.039 (3)	−0.013 (2)	0.002 (2)	0.003 (2)
N6	0.099 (4)	0.041 (3)	0.053 (3)	−0.016 (3)	0.010 (3)	0.006 (3)
O1	0.056 (2)	0.053 (2)	0.031 (2)	0.0045 (19)	0.0030 (17)	0.0068 (18)
O2	0.076 (3)	0.055 (3)	0.033 (2)	−0.007 (2)	−0.004 (2)	0.0133 (19)
S1	0.0669 (10)	0.0478 (9)	0.0364 (8)	0.0019 (7)	−0.0021 (7)	0.0085 (7)
S2	0.1025 (14)	0.0464 (9)	0.0316 (8)	−0.0095 (9)	−0.0022 (8)	0.0068 (7)
C1	0.051 (4)	0.041 (3)	0.043 (3)	0.002 (3)	0.004 (3)	−0.004 (3)
C2	0.045 (3)	0.042 (3)	0.034 (3)	−0.005 (2)	0.001 (2)	0.008 (2)
C3	0.043 (3)	0.039 (3)	0.042 (3)	−0.007 (2)	0.002 (2)	0.005 (2)
C4	0.053 (4)	0.065 (4)	0.035 (3)	−0.010 (3)	−0.001 (3)	0.005 (3)
C5	0.051 (4)	0.074 (4)	0.052 (4)	−0.011 (3)	−0.014 (3)	0.012 (3)
C6	0.044 (3)	0.057 (4)	0.060 (4)	−0.014 (3)	−0.003 (3)	0.018 (3)
C7	0.049 (3)	0.044 (3)	0.050 (4)	−0.006 (3)	0.003 (3)	0.010 (3)
C8	0.063 (4)	0.051 (4)	0.065 (5)	0.006 (3)	0.014 (3)	0.007 (3)
C9	0.071 (5)	0.055 (4)	0.086 (6)	0.008 (4)	0.022 (4)	0.015 (4)
C10	0.044 (4)	0.074 (5)	0.117 (7)	0.012 (3)	0.022 (4)	0.029 (5)
C11	0.046 (4)	0.075 (5)	0.088 (6)	−0.002 (3)	−0.004 (4)	0.027 (4)
C12	0.047 (3)	0.056 (4)	0.039 (3)	−0.003 (3)	−0.007 (3)	0.008 (3)
C13	0.049 (3)	0.033 (3)	0.050 (4)	−0.002 (2)	−0.011 (3)	0.005 (3)
C14	0.046 (4)	0.057 (4)	0.063 (4)	−0.002 (3)	0.002 (3)	0.001 (3)
C15	0.057 (4)	0.060 (4)	0.089 (6)	−0.015 (3)	−0.004 (4)	0.010 (4)
C16	0.104 (6)	0.073 (5)	0.083 (6)	−0.034 (5)	−0.039 (5)	0.005 (5)
C17	0.159 (9)	0.089 (6)	0.058 (5)	−0.054 (6)	−0.031 (6)	0.003 (5)
C18	0.114 (6)	0.073 (5)	0.045 (4)	−0.036 (4)	−0.017 (4)	0.007 (4)
C19	0.041 (3)	0.055 (4)	0.032 (3)	−0.009 (3)	−0.003 (2)	−0.002 (3)
C20	0.060 (4)	0.062 (4)	0.043 (4)	−0.012 (3)	0.001 (3)	−0.001 (3)
C21	0.080 (6)	0.099 (6)	0.054 (5)	−0.043 (5)	0.013 (4)	−0.009 (4)
C22	0.049 (5)	0.146 (9)	0.068 (6)	−0.030 (5)	0.012 (4)	−0.020 (6)
C23	0.044 (4)	0.128 (7)	0.073 (5)	0.015 (4)	−0.007 (4)	−0.007 (5)
C24	0.047 (4)	0.075 (5)	0.055 (4)	0.002 (3)	0.001 (3)	0.005 (3)
C25	0.055 (4)	0.053 (4)	0.038 (3)	−0.011 (3)	−0.003 (3)	0.000 (3)
C26	0.054 (4)	0.064 (4)	0.023 (3)	−0.022 (3)	−0.006 (2)	0.001 (3)
C27	0.076 (5)	0.061 (4)	0.030 (3)	−0.016 (3)	−0.016 (3)	0.009 (3)
C28	0.092 (6)	0.067 (5)	0.061 (5)	−0.029 (4)	−0.011 (4)	0.027 (4)
C29	0.099 (6)	0.103 (7)	0.052 (5)	−0.057 (5)	−0.009 (4)	0.033 (4)
C30	0.075 (5)	0.088 (5)	0.036 (4)	−0.035 (4)	−0.007 (3)	0.010 (4)
C31	0.059 (4)	0.080 (5)	0.030 (3)	−0.032 (4)	−0.010 (3)	0.000 (3)
C32	0.085 (6)	0.143 (8)	0.044 (5)	−0.059 (6)	0.000 (4)	0.018 (5)
C33	0.068 (6)	0.168 (10)	0.048 (5)	−0.044 (6)	0.001 (4)	0.000 (6)
C34	0.062 (5)	0.123 (7)	0.046 (4)	−0.015 (4)	−0.004 (3)	−0.014 (4)
C35	0.059 (4)	0.095 (6)	0.038 (4)	−0.020 (4)	−0.003 (3)	−0.004 (4)
C36	0.050 (3)	0.041 (3)	0.040 (3)	−0.002 (3)	−0.005 (3)	0.009 (3)
C37	0.052 (4)	0.039 (3)	0.046 (4)	−0.006 (3)	−0.007 (3)	−0.003 (3)
C38	0.062 (4)	0.076 (5)	0.051 (4)	−0.007 (3)	−0.009 (3)	0.009 (3)
C39	0.081 (6)	0.095 (6)	0.078 (6)	−0.003 (5)	−0.034 (5)	0.015 (5)
C40	0.067 (5)	0.083 (6)	0.100 (7)	0.003 (4)	−0.021 (5)	0.003 (5)
C41	0.066 (5)	0.082 (5)	0.073 (5)	−0.005 (4)	0.005 (4)	−0.007 (4)
C42	0.067 (5)	0.063 (4)	0.056 (4)	−0.004 (3)	−0.011 (3)	0.000 (3)
C43	0.048 (3)	0.036 (3)	0.043 (3)	−0.005 (2)	−0.003 (3)	0.001 (3)
C44	0.079 (5)	0.057 (4)	0.046 (4)	−0.014 (3)	−0.006 (3)	0.008 (3)
C45	0.099 (6)	0.057 (4)	0.047 (4)	−0.033 (4)	−0.001 (4)	0.005 (3)
C46	0.089 (5)	0.056 (4)	0.063 (5)	−0.025 (4)	−0.012 (4)	−0.002 (4)
C47	0.137 (7)	0.070 (5)	0.050 (4)	−0.035 (5)	−0.035 (5)	0.006 (4)
C48	0.104 (6)	0.054 (4)	0.054 (4)	−0.023 (4)	−0.022 (4)	0.015 (3)

### Geometric parameters (Å, °)

**Table:**

Sn1—O1	2.116 (4)	C17—H17	0.9300
Sn1—C19	2.128 (5)	C18—H18	0.9300
Sn1—C13	2.134 (5)	C19—C20	1.387 (8)
Sn1—N1	2.175 (4)	C19—C24	1.393 (8)
Sn1—S1	2.5420 (16)	C20—C21	1.400 (10)
Sn2—O2	2.088 (4)	C20—H20	0.9300
Sn2—C37	2.134 (6)	C21—C22	1.364 (11)
Sn2—C43	2.144 (5)	C21—H21	0.9300
Sn2—N4	2.185 (5)	C22—C23	1.369 (12)
Sn2—S2	2.5326 (16)	C22—H22	0.9300
N1—C1	1.299 (7)	C23—C24	1.379 (9)
N1—N2	1.401 (6)	C23—H23	0.9300
N2—C12	1.303 (7)	C24—H24	0.9300
N3—C12	1.371 (7)	C25—C26	1.440 (8)
N3—H3A	0.8600	C25—H25	0.9300
N3—H3B	0.8600	C26—C27	1.398 (9)
N4—C25	1.313 (7)	C26—C31	1.468 (9)
N4—N5	1.408 (6)	C27—C28	1.440 (9)
N5—C36	1.307 (7)	C28—C29	1.360 (11)
N6—C36	1.359 (7)	C28—H28	0.9300
N6—H6A	0.8600	C29—C30	1.400 (11)
N6—H6B	0.8600	C29—H29	0.9300
O1—C3	1.337 (6)	C30—C31	1.412 (9)
O2—C27	1.315 (8)	C30—C32	1.436 (11)
S1—C12	1.741 (6)	C31—C35	1.400 (9)
S2—C36	1.744 (6)	C32—C33	1.350 (12)
C1—C2	1.438 (8)	C32—H32	0.9300
C1—H1	0.9300	C33—C34	1.390 (12)
C2—C3	1.407 (7)	C33—H33	0.9300
C2—C7	1.442 (8)	C34—C35	1.380 (9)
C3—C4	1.423 (8)	C34—H34	0.9300
C4—C5	1.360 (8)	C35—H35	0.9300
C4—H4	0.9300	C37—C42	1.381 (9)
C5—C6	1.419 (9)	C37—C38	1.387 (8)
C5—H5	0.9300	C38—C39	1.380 (10)
C6—C11	1.399 (9)	C38—H38	0.9300
C6—C7	1.431 (8)	C39—C40	1.387 (11)
C7—C8	1.427 (8)	C39—H39	0.9300
C8—C9	1.360 (9)	C40—C41	1.367 (11)
C8—H8	0.9300	C40—H40	0.9300
C9—C10	1.383 (11)	C41—C42	1.391 (9)
C9—H9	0.9300	C41—H41	0.9300
C10—C11	1.379 (11)	C42—H42	0.9300
C10—H10	0.9300	C43—C44	1.374 (8)
C11—H11	0.9300	C43—C48	1.400 (8)
C13—C14	1.387 (8)	C44—C45	1.389 (9)
C13—C18	1.397 (9)	C44—H44	0.9300
C14—C15	1.392 (9)	C45—C46	1.362 (9)
C14—H14	0.9300	C45—H45	0.9300
C15—C16	1.387 (11)	C46—C47	1.371 (10)
C15—H15	0.9300	C46—H46	0.9300
C16—C17	1.341 (11)	C47—C48	1.378 (9)
C16—H16	0.9300	C47—H47	0.9300
C17—C18	1.385 (10)	C48—H48	0.9300
			
O1—Sn1—C19	93.23 (19)	C13—C18—H18	119.9
O1—Sn1—C13	92.32 (19)	C20—C19—C24	118.3 (6)
C19—Sn1—C13	122.7 (2)	C20—C19—Sn1	122.1 (4)
O1—Sn1—N1	81.88 (15)	C24—C19—Sn1	119.6 (4)
C19—Sn1—N1	111.26 (18)	C19—C20—C21	119.9 (7)
C13—Sn1—N1	126.00 (19)	C19—C20—H20	120.1
O1—Sn1—S1	157.99 (11)	C21—C20—H20	120.1
C19—Sn1—S1	98.65 (16)	C22—C21—C20	120.0 (7)
C13—Sn1—S1	96.69 (16)	C22—C21—H21	120.0
N1—Sn1—S1	76.57 (12)	C20—C21—H21	120.0
O2—Sn2—C37	91.4 (2)	C21—C22—C23	121.2 (7)
O2—Sn2—C43	93.16 (19)	C21—C22—H22	119.4
C37—Sn2—C43	118.6 (2)	C23—C22—H22	119.4
O2—Sn2—N4	81.15 (16)	C22—C23—C24	119.0 (8)
C37—Sn2—N4	115.57 (18)	C22—C23—H23	120.5
C43—Sn2—N4	125.61 (18)	C24—C23—H23	120.5
O2—Sn2—S2	157.71 (13)	C23—C24—C19	121.6 (7)
C37—Sn2—S2	101.39 (17)	C23—C24—H24	119.2
C43—Sn2—S2	96.54 (16)	C19—C24—H24	119.2
N4—Sn2—S2	76.88 (12)	N4—C25—C26	126.2 (6)
C1—N1—N2	114.9 (5)	N4—C25—H25	116.9
C1—N1—Sn1	126.3 (4)	C26—C25—H25	116.9
N2—N1—Sn1	118.6 (3)	C27—C26—C25	121.2 (6)
C12—N2—N1	114.2 (5)	C27—C26—C31	120.8 (6)
C12—N3—H3A	120.0	C25—C26—C31	118.1 (6)
C12—N3—H3B	120.0	O2—C27—C26	124.3 (6)
H3A—N3—H3B	120.0	O2—C27—C28	117.3 (6)
C25—N4—N5	114.2 (5)	C26—C27—C28	118.4 (7)
C25—N4—Sn2	126.1 (4)	C29—C28—C27	120.6 (8)
N5—N4—Sn2	119.6 (3)	C29—C28—H28	119.7
C36—N5—N4	114.6 (4)	C27—C28—H28	119.7
C36—N6—H6A	120.0	C28—C29—C30	122.5 (7)
C36—N6—H6B	120.0	C28—C29—H29	118.8
H6A—N6—H6B	120.0	C30—C29—H29	118.8
C3—O1—Sn1	125.5 (3)	C29—C30—C31	119.9 (7)
C27—O2—Sn2	126.7 (4)	C29—C30—C32	121.0 (8)
C12—S1—Sn1	92.4 (2)	C31—C30—C32	119.1 (8)
C36—S2—Sn2	93.7 (2)	C35—C31—C30	117.6 (6)
N1—C1—C2	126.7 (5)	C35—C31—C26	124.5 (6)
N1—C1—H1	116.7	C30—C31—C26	117.9 (7)
C2—C1—H1	116.7	C33—C32—C30	121.0 (8)
C3—C2—C1	122.1 (5)	C33—C32—H32	119.5
C3—C2—C7	119.4 (5)	C30—C32—H32	119.5
C1—C2—C7	118.5 (5)	C32—C33—C34	120.1 (8)
O1—C3—C2	123.1 (5)	C32—C33—H33	119.9
O1—C3—C4	117.1 (5)	C34—C33—H33	119.9
C2—C3—C4	119.8 (5)	C35—C34—C33	120.1 (9)
C5—C4—C3	120.6 (6)	C35—C34—H34	120.0
C5—C4—H4	119.7	C33—C34—H34	120.0
C3—C4—H4	119.7	C34—C35—C31	122.1 (7)
C4—C5—C6	122.4 (6)	C34—C35—H35	119.0
C4—C5—H5	118.8	C31—C35—H35	119.0
C6—C5—H5	118.8	N5—C36—N6	117.8 (5)
C11—C6—C5	121.9 (6)	N5—C36—S2	127.1 (4)
C11—C6—C7	120.1 (6)	N6—C36—S2	115.1 (4)
C5—C6—C7	118.1 (6)	C42—C37—C38	117.0 (6)
C8—C7—C6	116.5 (6)	C42—C37—Sn2	121.3 (5)
C8—C7—C2	123.7 (6)	C38—C37—Sn2	121.4 (5)
C6—C7—C2	119.7 (5)	C39—C38—C37	121.5 (7)
C9—C8—C7	121.4 (7)	C39—C38—H38	119.2
C9—C8—H8	119.3	C37—C38—H38	119.2
C7—C8—H8	119.3	C38—C39—C40	120.4 (7)
C8—C9—C10	121.7 (7)	C38—C39—H39	119.8
C8—C9—H9	119.2	C40—C39—H39	119.8
C10—C9—H9	119.2	C41—C40—C39	119.1 (8)
C11—C10—C9	119.2 (7)	C41—C40—H40	120.4
C11—C10—H10	120.4	C39—C40—H40	120.4
C9—C10—H10	120.4	C40—C41—C42	119.9 (8)
C10—C11—C6	121.2 (7)	C40—C41—H41	120.0
C10—C11—H11	119.4	C42—C41—H41	120.0
C6—C11—H11	119.4	C37—C42—C41	122.1 (7)
N2—C12—N3	117.0 (5)	C37—C42—H42	119.0
N2—C12—S1	127.3 (4)	C41—C42—H42	119.0
N3—C12—S1	115.6 (5)	C44—C43—C48	117.5 (5)
C14—C13—C18	117.9 (6)	C44—C43—Sn2	121.5 (4)
C14—C13—Sn1	122.7 (4)	C48—C43—Sn2	120.8 (4)
C18—C13—Sn1	119.3 (5)	C43—C44—C45	121.0 (6)
C13—C14—C15	120.9 (6)	C43—C44—H44	119.5
C13—C14—H14	119.5	C45—C44—H44	119.5
C15—C14—H14	119.5	C46—C45—C44	121.1 (6)
C16—C15—C14	119.5 (7)	C46—C45—H45	119.5
C16—C15—H15	120.2	C44—C45—H45	119.5
C14—C15—H15	120.2	C45—C46—C47	118.8 (6)
C17—C16—C15	120.0 (7)	C45—C46—H46	120.6
C17—C16—H16	120.0	C47—C46—H46	120.6
C15—C16—H16	120.0	C46—C47—C48	121.0 (7)
C16—C17—C18	121.4 (8)	C46—C47—H47	119.5
C16—C17—H17	119.3	C48—C47—H47	119.5
C18—C17—H17	119.3	C47—C48—C43	120.7 (6)
C17—C18—C13	120.2 (7)	C47—C48—H48	119.7
C17—C18—H18	119.9	C43—C48—H48	119.7

### Hydrogen-bond geometry (Å, °)

**Table:**

*D*—H···*A*	*D*—H	H···*A*	*D*···*A*	*D*—H···*A*
N6—H6B···O1^i^	0.86	2.35	3.209 (6)	177
N3—H3A···S2^ii^	0.86	2.87	3.522 (6)	134

Symmetry codes: (i) *x*−1, *y*−1, *z*; (ii) −*x*+1, −*y*+1, −*z*+1.

## References

[bb1] Bruker (2001). *SADABS* Bruker AXS Inc., Madison, Wisconsin, USA.

[bb2] Bruker (2007). *SMART* and *SAINT* Bruker AXS Inc., Madison, Wisconsin, USA.

[bb3] Sarma, M. S., Mazumder, S., Ghosh, D., Roy, A., Duthie, A. & Tiekink, E. R. T. (2007). *Appl. Organomet. Chem.* **21**, 890–905.

[bb4] Sheldrick, G. M. (2008). *Acta Cryst.* A**64**, 112–122. 10.1107/S0108767307043930 18156677

